# Antimicrobial Peptides with Antibacterial Activity against Vancomycin-Resistant *Staphylococcus aureus* Strains: Classification, Structures, and Mechanisms of Action

**DOI:** 10.3390/ijms22157927

**Published:** 2021-07-25

**Authors:** Isabella Hernández-Aristizábal, Iván Darío Ocampo-Ibáñez

**Affiliations:** Research Group of Microbiology, Industry and Environment, Faculty of Basic Sciences, Universidad Santiago de Cali, Cali 760035, Colombia; isabella.hernandez00@usc.edu.co

**Keywords:** antimicrobial peptides, vancomycin-resistant *Staphylococcus aureus*, vancomycin-intermediate resistant *Staphylococcus aureus*

## Abstract

The emergence of bacteria resistant to conventional antibiotics is of great concern in modern medicine because it renders ineffectiveness of the current empirical antibiotic therapies. Infections caused by vancomycin-resistant *Staphylococcus aureus* (VRSA) and vancomycin-intermediate *S. aureus* (VISA) strains represent a serious threat to global health due to their considerable morbidity and mortality rates. Therefore, there is an urgent need of research and development of new antimicrobial alternatives against these bacteria. In this context, the use of antimicrobial peptides (AMPs) is considered a promising alternative therapeutic strategy to control resistant strains. Therefore, a wide number of natural, artificial, and synthetic AMPs have been evaluated against VRSA and VISA strains, with great potential for clinical application. In this regard, we aimed to present a comprehensive and systematic review of research findings on AMPs that have shown antibacterial activity against vancomycin-resistant and vancomycin-intermediate resistant strains and clinical isolates of *S. aureus*, discussing their classification and origin, physicochemical and structural characteristics, and possible action mechanisms. This is the first review that includes all peptides that have shown antibacterial activity against VRSA and VISA strains exclusively.

## 1. Introduction

The emergence of bacterial resistance (BR) is one of the most critical public health concerns in recent years. The rapid spread of resistant bacteria compromises the efficacy of antibiotic treatments and has serious implications in the practice of modern medicine [1]. Historically, antibiotics have been used to treat bacterial infections. However, some factors, such as their indiscriminate prescription, inappropriate use in the food industry, lack of discovery of new antibiotics, and poor quality of available antibiotics, have accelerated the emergence of BR [2]. Consequently, BR causes high morbidity and mortality rates, significant increase in healthcare costs, and use of antibacterial agents with increased host toxicity [3,4]. In this context, both Gram-negative and Gram-positive bacteria can be resistant to conventional antibiotics, which limits the number of antimicrobial agents that can be effectively used against these bacterial groups [5]. In particular, BR in Gram-positive species presents a worrisome scenario, since several species show multiple drug resistance and cannot be controlled with conventional antibiotics, leading to the use of last-line drugs in higher concentrations, which can have toxic effects on the patients’ health [6]. Due to the concerns associated with BR and its serious impact on global public health, the World Health Organization (WHO) has recently published a list of priority bacteria resistant to antibiotics [7], through which WHO seeks to guide and promote research and development of new alternatives to control resistant bacteria [2]. The list includes different species of Gram-positive bacteria that cause important community and nosocomial infections, including methicillin-resistant *S. aureus* (MRSA) and vancomycin-resistant *S. aureus* (VRSA) [7,8]. *S. aureus* is a bacterium that is frequently isolated in hospital and community settings, causing various skin and soft tissue infections, as well as severe bone and joint infections. It can also cause endocarditis; bacteremia; and, in more severe cases, toxic shock syndrome and death [9].

Initially, MRSA strains emerged after the introduction of methicillin in 1959 and were only associated with hospital settings. However, these strains that have now widely spread around the world are known to be community-associated MRSA strains with wide genetic diversity, easy transmission, and increased virulence [10]. The evolution of MRSA has been mostly framed in hospital settings, where clonal spread occurs easily from one patient to another and sometimes through healthcare personnel [10]. Methicillin resistance is caused due to the acquisition of mobile chromosomal element known as Staphylococcal cassette chromosome mec (SCCmec) by methicillin-susceptible *S. aureus* (MSSA) strains [11]. Acquisition of this chromosomal fragment generates an expression of a new penicillin-binding protein (PBP2a) having low affinity for beta-lactams [4]. Initially, MRSA isolates had resistance to only one class of antibiotics; however, nowadays they have multi-antibiotic resistance, including resistance to vancomycin. This generates a serious public health issue since vancomycin is the last line of treatment against infections caused due to resistant strains of *S. aureus* [12,13,14]. In this regard, resistance to vancomycin causes high mortality rates and increases the risk of premature death when compared with infections caused by susceptible strains, as it increases the length of hospital stay [15,16]. Vancomycin resistance by VRSA strains has been associated with the acquisition of van genes (vanA, vanB, vanC, vanD, vanE, vanF), which generate a low affinity for some glycopeptide antibiotics [17]. However, antibiotics such as oritavacin, a semisynthetic glycopeptide [18], and corbomycin and complestatin, which belong to the type V family of glycopeptides [19], have shown activity against MRSA and VRSA strains. These glycopeptides have several mechanisms of action against cell wall of *S. aureus*, including the inhibition of peptidoglycan synthesis and the inhibition of fatty acid synthesis [18,19]. Despite these alternatives, resistant strains of *S. aureus* can have a wide and diverse variety of resistance mechanisms, hindering their control with the use of currently available conventional antibiotics for the treatment of the infections caused by them [12]. In view of this situation, it is crucial to search and develop new antimicrobial alternatives to combat resistant *S. aureus* strains, especially VRSA strains, which cause significant concern in terms of global public health [7].

In this regard, antimicrobial peptides (AMPs) are a promising alternative to conventional antibiotics because of their great potential to combat resistant bacteria [20]. From a pharmacodynamic point of view, AMPs can have a much higher death rate than antibiotics, even against resistant strains [21]. AMPs are naturally produced small molecules that are part of the innate immune system of different organisms as an effective defense against infections caused by bacteria, fungi, viruses, and some protozoa [22]. Although AMPs are widely diverse, they share common characteristics, such as size (generally between 12 and 50 amino acids) and 3D structures [23]. However, they can differ greatly in terms of amino acid content, activity, targets, action mechanisms, origin, and physicochemical properties [22,24]. According to their activity, AMPs can be classified as antibacterial, antifungal, antiviral, or antiparasitic peptides [25]. The most studied type are the antibacterial AMPs, which are diverse, have different physicochemical properties and can have widely diverse structures, which plays a fundamental role in their biological activity [25]. Antibacterial AMPs have a wide range of action mechanisms and can act on different molecular targets within the bacterial cells, for example, by inducing damage to the bacterial membrane or by inhibiting the synthesis of proteins, enzymes, and nucleic acids at the cytoplasmic level, as well as affecting protein folding [25,26,27]. Because of these characteristics, AMPs have a great potential in the control of bacteria susceptible and resistant to conventional antibiotics that are responsible for infections affecting human health. In this regard, several groups of AMPs have shown high efficacy against bacteria and other pathogens, including strains and clinical isolates of VRSA and vancomycin-intermediate *S. aureus* (VISA) [28,29]. There is a continuous development in the field of research on peptide activity, their possible molecular targets, and their possible action mechanisms against this particular type of bacterial isolate. The purpose of this review is to comprehensively and systematically describe research findings on AMPs that have shown antibacterial activity against VRSA and VISA strains and clinical isolates, discussing their classification, structure, and possible action mechanisms. This is the first review that collects and classifies all peptides that have shown antibacterial activity against VRSA and VISA strains exclusively.

## 2. Phenotypic and Genotypic Characteristics of VRSA and VISA Strains That Showed Susceptibility to AMPs

Infections caused by *S. aureus* are treated with conventional antibiotics that are effective against susceptible strains. However, this efficacy is reduced in the case of resistant strains [30]. Nowadays, a wide diversity of strains and clinical isolates of *S. aureus* have been reported to show resistance to different antibiotics and contain a wide range of genes in their genomes that make them resistant to antibiotics [10,31]. In light of this situation, AMPs appear as promising alternatives to control this type of bacteria. However, the emergence of strains resistant to AMPs has recently been reported, although it is believed that this resistance is much less likely to evolve than the resistance to conventional antibiotics, and it is believed to occur more easily within in vitro systems than in vivo [21,32,33]. Considering this, it is important to identify the phenotypic and genotypic characteristics of the strains that show susceptibility to AMPs in order to provide relevant information to study resistance to AMPs. There is a scarcity of reports that include genotypic and phenotypic characterization of strains and clinical isolates. Table 1 summarizes the profiles for susceptibility and resistance to conventional antibiotics, as well as the resistance genes identified in VRSA and VISA strains that were evaluated against the AMPs included in this review.

Regarding the phenotypic characterization of these strains, the susceptibility and resistance profiles included seven antimicrobial categories, namely, beta-lactams (penicillin, amoxicillin, and oxacillin), macrolides (erythromycin), glycopeptides (vancomycin), tetracyclines (tetracycline), lipopeptides (daptomycin), oxazolidinones (linezolid), and lincosamides (clindamycin) (Table 1). Susceptibility to these antibiotics was evaluated by broth microdilution or disk diffusion, according to the protocols established by the Clinical and Laboratory Standards Institute (CLSI) and the European Committee on Antimicrobial Susceptibility Testing (Table 1). Taking this into account, strains with phenotypic characterization showed resistance to at least one antibiotic from the seven antimicrobial categories mentioned above (Table 1). Thus, a total of 66 strains and clinical isolates with different phenotypic profiles were identified. In this regard, 33 strains were identified as VRSA and 33 as VISA, as they showed resistance and intermediate resistance to vancomycin, respectively (Table 1). Six of these strains can be considered multidrug-resistant, as they showed resistance to at least three different antimicrobial categories, including combined resistance to beta-lactams, macrolides, glycopeptides, tetracyclines, and lincosamides (Table 1). These results are consistent with the numerous reports on the dissemination of VRSA and VISA strains in recent years, especially with the high spread of vancomycin-resistant MRSA strains that represent a serious threat to human health due to the ineffectiveness of conventional antibiotic therapies [61,62,63,64].

Regarding genotypic characterization, there is a dearth of studies that report the presence of resistance genes in the strains tested against AMPs (Table 1). This suggests that most studies do not take into account the presence of resistance-related genetic factors in the strains tested against antimicrobial peptides. A total of two vancomycin-resistant strains had the transposon-like heterogeneous mobile chromosomal element known as SCCmec type II in their genome: one VISA and one VRSA strain; additionally, one VRSA strain had the mecA gene (Table 1). Although these genetic characteristics have been reported in MRSA strains, VRSA and VISA strains with these genetic mechanisms have already been reported [65]. SCCmec type II is related to MRSA clones associated with hospital settings, which could be related to prolonged vancomycin treatment [65,66]. In particular, the mecA gene is responsible for methicillin resistance and can be acquired by susceptible strains through horizontal transfer mediated by SCCmec, which is integrated into the chromosome of strains associated with hospital and community environments [67,68]. SCCmec works as a genetic exchange vehicle for staphylococcal species, in particular as a mechanism of adaptation to environmental conditions including antibiotic selective pressure [69]. The mecA gene codes for the PBP2a protein with a low affinity for beta-lactam antibiotics [70]. PBP2a replaces all other penicillin-binding proteins and provides broad resistance to all beta-lactam antibiotics, which may include penicillins, cephalosporins, and carbapenemics [10]. In addition, it has been documented that the SCCmec element may contain other types of genes resistant to mercury, cadmium, kanamycin, bleomycin, erythromycin, spectinomycin, and fusidic acid, among other categories of antibiotics [71,72]. In this regard, strains containing SCCmec type II showed resistance to vancomycin, daptomycin, and clindamycin (Table 1). On the other hand, the vanA gene was identified in a clinical VRSA isolate. In addition, this strain showed resistance to other antibiotics, such as penicillin, amoxicillin, and erythromycin (Table 1). For some decades now, glycopeptide antibiotics, such as vancomycin, have become an ideal option for treating infections caused by *S. aureus* strains resistant to multiple antibiotics [17,73,74]. However, strains with resistance and intermediate resistance to vancomycin have been reported during the last few years [73]. VRSA strains are mainly associated with long periods of hospitalization, persistent infections, or failed treatments [75]. Vancomycin interferes with peptidoglycan synthesis by forming non-covalent bonds with d-Ala-d-Ala residues, disrupting bacterial cell wall assembly [76]. Vancomycin resistance is mediated by van genes, which control the substitution of the d-Ala-d-Ala terminus of the peptidoglycan monomer [17,75]. These genes were first found in *Enterococcus* spp. and were transmitted to other bacterial species, such as *S. aureus* [17]. To date, 11 van genes are known, which are classified as (1) genes that mediate the substitution of the d-Ala-d-Ala terminus of the peptidoglycan by d-Ala-d-Lactate, such as vanA, vanB, vanD, vanF, vanI, and vanM, which generate high-level resistance, and (2) genes responsible for the substitution of d-Ala-d-Ala by d-Ala-d-Ser, including vanC, vanE, vanG, vanL, and vanN, associated with low-level resistance [75]. Finally, two strains of VISA had the graS gene (Table 1). In particular, VISA strains, despite not having the van genes in their genome, express a resistance phenotype related to a thickening of the cell wall that occurs due to prolonged exposure to vancomycin and results in an increased amount of antibiotic needed for its control [77]. The reasons why VISA strains show intermediate resistance are not known yet, although genetically these strains contain multiple mutations in genes related to cell wall-associated proteins [76]. In this regard, mutations in the graS gene have been found to be associated with the occurrence of VISA isolates [51]. Overexpression of this gene in VISA strains results in an increase in vancomycin MIC and the expression of some genes such as those involved in cell wall synthesis [78]. In addition, the mutant strains with the graS gene show greater sensitization to AMPs [79]. None of the genes reported for VRSA and VISA strains (Table 1) have previously been associated with resistance to AMPs.

Although vancomycin-resistant isolates have not spread as successfully as MRSA, their grade of resistance and their strong clinical impact have increased over time. Therefore, it is necessary to search for new antimicrobials to control VRSA and VISA strains with wide genetic and phenotypic diversity. AMPs are considered promising alternatives for this purpose [65].

## 3. Classification of AMPs with Antibacterial Activity against VRSA and VISA Strains

AMPs with antimicrobial activity have been found and isolated from different secretions, cells, or in many tissues of different organisms, including plants and animals [80]. AMPs have a wide range of comparative advantages over conventional antibiotics due to their ability to interact with bacterial membranes via electrostatic interactions, penetrate cells affecting cellular functions causing bacterial death, and act on a wide spectrum of bacteria and be less likely to induce resistance [80]. Additionally, AMPs can be used in combination with conventional antibiotics, with highly positive synergistic effects that can help fight serious infectious diseases caused even by resistant bacteria [81]. Despite all these advantages, AMPs can have some disadvantages. For example, some naturally occurring peptides can be easily degraded or have strong cytotoxic and hemolytic effects on human cells; hence, it is necessary to optimize and substantially modify sequences in some of their residues to reduce such adverse effects [82]. Thus, peptides with specific or artificial modifications can be developed through bioinformatics strategies or in a laboratory with high production cost, but also with the possibility of reducing their negative effects and improving their clinical application [80,82]. Therefore, there is a wide diversity of antimicrobial peptides, including natural and artificial or modified peptides, which can be produced in a laboratory. Despite this wide diversity, both natural and artificial AMPs that have shown activity against VRSA and VISA strains and clinical isolates can be classified according to (1) organism of origin, (2) structural characteristics, and (3) mechanism of action. A total of 66 AMPs reported in literature showed antibacterial activity against VRSA and VISA strains and clinical isolates (Table 2, Table 3 and Table 4).

### 3.1. AMP Classification Based on Their Origin

AMPs act as a defense mechanism against invading cells in animals, plants, fungi, and microorganisms [80]. The immune system of most organisms has primary reaction mechanisms using AMPs to target a specific class of microorganisms through rapid and lethal action mechanisms [83]. Particularly in animals, AMPs trigger different defense mechanisms according to their interactions with the environment in which they live [83]. In this context, it is known that the highest concentrations of these antimicrobial molecules are generally found in epithelial tissues frequently exposed to pathogens or in cells involved in host defense [83]. Thus, some body fluids, such as blood, sweat, saliva, plasma, white blood cell secretions, and granule extracts, have been extensively studied for their antimicrobial characteristics [84,85,86]. A wide variety of AMPs from various animal species have been found to show high antimicrobial capacity against bacteria susceptible and resistant to conventional antibiotics [87]. In particular, AMPs that have shown antibacterial activity against VRSA and VISA strains have been isolated from arthropods, amphibians, mammals, and bacteria. Even artificial AMPs have shown activity against these types of resistant strains.

#### 3.1.1. Animal-Derived AMPs

A total of 28 animal-derived AMPs reported in the literature showed antibacterial activity against vancomycin-resistant *S. aureus* strains. Table 2 lists all animal-derived AMPs that have demonstrated antimicrobial activity against VRSA and VISA strains. There has been extensive progress in the studies on AMPs obtained from invertebrate animals, and in this regard, a large proportion of AMPs with activity against these strains have been isolated and identified in arthropods [88]. A wide variety of natural insect-produced peptides and their analogs with antibacterial, antifungal, and antiviral activity are now known [88]. Insects, which constitute the largest class of animals on earth, accounting for about 50% of all known species, have simple but well-developed immune systems with a wide arsenal of bioactive molecules, including AMPs [89]. Among the best-known families of insect-derived antimicrobial peptides are the melittins and defensins. Melittin is a peptide isolated from the venom of the *Apis mellifera* bee that has been extensively studied and has shown bactericidal activity against resistant *S. aureus* strains [90]. This AMP exhibited potent antibacterial activity against VISA clinical isolates, with an MIC of 2 μM and an MBC of 4 μM [50]. Despite its bactericidal effect, melittin has shown strong cytotoxic and hemolytic activity. As a result, a wide variety of peptide analogs have been designed, synthesized, and tested on the basis of melittin, such as the Hec peptide [91]. This AMP showed antibacterial activity against Gram-positive bacteria, including VRSA strains (MIC > 80 μM), and its toxic effect was detected only at very high concentrations [35]. In addition, when Hec was tested in combination with vancomycin, its activity was significantly enhanced. However, a high toxic effect on epithelial cells was observed [35,92]. The defensin family includes AMPs that effectively combat Gram-positive bacteria and can be found in various insect species, including diptera [88]. Recently, formicin C was identified in the house fly *Musca domestica*, a type of defensin that was shown to effectively combat wounds infected with resistant strains of *S. aureus* in an in vivo model of *Hermetia illucens* larvae [46]. Formicin C successfully inhibited the growth of vancomycin-resistant MRSA strains, with MIC of 32 μg/mL [46]. More specifically, this peptide was able to negatively affect the expression of genes with a significant role in the formation of biofilms by resistant strains of *S. aureus* [46]. On the other hand, the AMP cecropin A identified in the giant silk moth *Hyalophora cecropia* showed broad-spectrum activity against resistant bacteria, including VISA strains [93]. In in vitro assays, this peptide showed a minimum inhibitory concentration of 64 μg/mL against the growth of VISA strains and caused a low toxic effect in human cells [49]. In addition, when murine models were intravenously infected with VISA strains and treated with this peptide, a 60% reduction in mortality was observed [49]. Finally, several AMPs with antimicrobial activity against Gram-positive bacteria have been identified in wasp [48,94]. In this respect, agelaia-MPI and protonectin are venom-derived peptides isolated from two species of wasps, *Parachartergus fraternus* and *Agelaia pallipes pallipes,* which showed antimicrobial activity against vancomycin-resistant *S. aureus* strains [48]. Agelaia-MPI is a peptide highly hemolytic that exhibited a potent antibacterial effect against VRSA strains (MIC between 4 and 8 μg/mL) [48]. Despite its moderated hemolytic effect against human erythrocytes, protonectin showed antibacterial activity against VRSA strains (MIC = 16 μg/mL) [48]. NeuroVAL and protonectin-F, analogues peptides of agelaia-MPI and protonectin, respectively, were designed to reduce nonspecific toxicity and improve potency [48]. Despite its reduced toxic effect on eukaryotic cells, NeuroVAL showed higher inhibitory concentration against VRSA strains (MIC > 128 μg/mL) compared to the canonical agelaia-MPI peptide [48]. The antimicrobial activity against VRSA strains and the toxic effect on cancerous and non-cancerous cell lines were very similar between protonectin-F and the canonical protonectin [48]. 

Arachnids are another group of arthropods that attract great interest because they are a rich source of molecules with promising characteristics for drug therapy. The venom of these animals has shown a cocktail of AMPs with antimicrobial characteristics with potential to combat bacteria that are resistant to conventional antibiotics [95]. In this sense, AMP ctriporin has been identified in the venom of the scorpion *Chaerilus tricostatus*, which showed inhibitory activity on the growth of resistant Gram-positive bacterial strains, such as VRSA, VISA, methicillin-resistant coagulase-negative *Staphylococcus*, and penicillin-resistant *Staphylococcus epidermis* [29]. In vitro and in vivo experiments carried out with this peptide showed an MIC of 10 μg/mL against both VRSA and VISA strains, as well as a significantly positive skin recovery effect in rabbits [29]. The AMP Smp24 isolated from the venom of the North African scorpion *Scorpio maurus palmatus* has shown broad activity against Gram-negative and Gram-positive bacteria [54]. In particular, this peptide showed an antibacterial effect against VISA strains (MIC between 32 and 64 μg/mL), with a low hemolytic effect against sheep erythrocytes [54]. On the other hand, it has been established that some ectoparasites, which are vectors of animal diseases, have immune systems with arsenals of defense molecules rich in AMPs [96]. Persulcatusin (PI) was found in the midgut of the tick *Ixodes persulcatus*, a peptide that showed MIC between 2 and 8 μg/mL against VRSA and VISA strains [34]. Similarly, the IR peptide derived from *Ixodes ricinus* showed activity against VISA (MIC > 32 μg/mL) and VRSA (MIC = 32 μg/mL) strains [97]. HAE and OMBAC peptides identified in the tick species *Haemaphysalis longicornis* and *Ornithodoros moubata*, respectively, also showed antibacterial activity against resistant *S. aureus* strains [34]. The inhibitory concentrations for HAE against VISA and VRSA (MIC > 32 μg/mL) were comparable to those found for OMBAC against these same strains (MIC > 32 μg/mL for VISA and MIC = 8 μg/mL for VRSA) [34].

Vertebrate animals have complex and well-developed defense mechanisms that protect them from invading pathogens. Amphibians have a rich chemical arsenal in their skin, including a great diversity of AMPs [98]. Amphibian skin provides protection from external agents and also performs a variety of functions including respiration, osmoregulation, and thermoregulation [98]. Many amphibian-derived AMPs have demonstrated antimicrobial activity against VRSA and VISA strains and clinical isolates (Table 2). Magainins, including magainin-1 and -2, are a family of AMPs isolated from the skin of the African frog *Xenopus laevis* belonging to the *Pipidae* family, which have demonstrated antimicrobial activity against fungi, protozoa, and Gram-positive and Gram-negative bacteria [99]. Magainin-2 has been extensively studied and it possesses activity against Gram-positive bacteria and has a low hemolytic effect [98]. Magainin-2 exhibited potent activity against VISA strains, with inhibitory concentrations of 16 μg/mL [49]. A 50% reduction in mortality was observed when murine models were intravenously infected with VISA strains and treated with this peptide [49]. Additionally, temporins are a large family of AMPs identified and isolated from frog skin with antibacterial activity against Gram-positive bacteria [100]. In particular, temporin-CPa and temporin-CPb from *Lithobates capito*, showed moderate activity against VISA strains with MIC of > 25 μM and 12.5 μM, respectively, and low hemolytic effect on human erythrocytes [56]. Temporin-1SPa from *Rana septentrionalis* showed activity against VISA strains (MIC = 12.5 μM) and moderate hemolytic activity [56]. Temporin-1Oc from *Rana ornativentris*, temporin-1Ga from *Rana grylio*, and temporin-1OLa from *Rana okaloosae* showed potent antimicrobial activity against VISA strain Mu50 (MIC of 1.6 μM, 6.2 μM, and 3.1 μM, respectively), but these AMPs showed a strong hemolysis against human red blood cells, with hemolytic concentrations between 12.5 and 50 μM [56]. Finally, fallaxin isolated from *Leptodactylus fallax*, is another amphibian-derived AMPs that has shown antimicrobial activity against Gram-negative bacteria exclusively [60]. A total of 65 analog peptides of fallaxin were designed through rational substitution of amino acids in the canonical sequence, and then tested for hemolytic activity and antibacterial activity against Gram-positive bacteria [60]. In this respect, the analogs FL9, FL10, FA12, and FL14 showed the lowest inhibitory concentrations against VISA strains (MIC values of 50 μM); however, they showed the highest hemolytic activity [60]. 

Mammalian skin, organ epithelium, blood, and saliva store different cellular and molecular components, including AMPs, which provide a defense mechanism against potential pathogens [101]. Mammalian-derived peptides have great potential to combat bacterial infections caused by resistant strains of *S. aureus*, including VRSA and VISA strains (Table 2). Cathelicidins are among the best-known mammalian-derived AMPs and have strong antibacterial activity [102]. There are different cathelicidins identified in many mammalian species, among which the LL-37 peptide stands out [41]. This AMP is a human cathelicidin identified in neutrophils that has shown broad-spectrum in vitro activity against virus and Gram-negative and Gram-positive bacteria, including VRSA and VISA strains (MIC = 64 μg/mL), as well as a low cytotoxic effect [41,103,104]. LL-13 and LL-17 are shorter peptides, derived from fragments of the canonical sequence of LL-37, which showed activity against VRSA strains [41]. Both LL-13 and LL-17 showed high inhibitory concentrations against VRSA and VISA strains compared to the canonical LL-37 peptide (Table 2) [41].

#### 3.1.2. Bacteria-Derived AMPs

Diverse interactions occur naturally between bacterial species sharing the same habitat, which are determined by the nutritional resources available [105]. Through various mechanisms, such as the production of toxic molecules or compounds, many bacterial species can favor their own survival and evolution, affecting other bacterial species they live with [105]. One of these mechanisms involves peptides that may be naturally produced by some bacterial species to control the survival of other bacteria [105]. Due to their strong effect, some bacterial-derived AMPs have been evaluated as alternatives to control Gram-positive bacteria resistant to conventional antibiotics [106]. In this regard, numerous AMPs identified in bacteria have shown promising characteristics against resistant VRSA and VISA strains (Table 3). Depending on the biosynthetic route they use, bacterial-derived peptides can be classified into two groups: (1) ribosomally synthesized peptides such as bacteriocins and (2) non-ribosomal peptides, such as bacitracins and glycopeptides [107]. Bacteriocins are a group of AMPs with a wide variety in size, structure, and mode of action [108]. Bacteriocins derived from Gram-positive bacteria can be grouped into four different classes: (I) lantibiotics, (II) non-lantibiotics, (III) large peptides, and (IV) bacteriocins containing lipids or carbohydrates [108]. Within the lantibiotics, two subclasses are identified: subclass Ia, which includes AMPs such as nisin, hominicin, and mutancin 1140, and subclass Ib, which includes mersacidin [108]. One of the best known bacteriocins is nisin derived from *Lactococcus lactis* [107]. This AMP has a strong antimicrobial effect, and according to in vitro assays, it showed activity against VISA strains, with MIC between 4.1 and 8.3 μg/mL, and a slight hemolytic effect against sheep erythrocytes [53,109]. Similarly, hominicin produced by *Staphylococcus hominis* has shown activity against Gram-positive bacteria [52]. This AMP showed a strong antibacterial effect against VISA strains (MIC = 3.82 μg/mL) in antimicrobial assays [52]. The mutancin 1140 AMP derived from *Streptococcus mutans* has been widely studied and showed strong activity against Gram-positive-resistant strains [110,111]. In particular, this peptide showed activity against VRSA and VISA strains, with MIC ranging from 4 to 8 μg/mL [44]. In addition, mutancin 1140 sensitization tests have shown that no BR to this AMP has been generated [44]. On the other hand, mersacidin is an anionic AMP that has successfully inhibited the in vitro growth of *S. aureus*; more specifically, it showed antimicrobial activity against resistant VISA-type strains (MIC = 35 μg/mL) [55,112]. Non-lantibiotic AMPs are classified into four subclasses: IIa, IIb, IIc, and IId [108]. Within subclass IId, we recognize bactofencin A, which is a short AMP derived from *Lactobacillus salivarius* isolated from the pig intestine. This AMP inhibits the growth of clinically significant pathogens [28]. Bactofencin A showed very strong activity against Gram-positive bacteria; specifically, analog 5 showed an antibacterial effect against VRSA strains isolated from bovine mastitis (MIC between 4.3 μM and 100 μM) but did not show activity against *Enterococcus fecalis* and *Streptococcus pyogenes* [28]. Additionally, non-ribosomally synthesized peptides from bacteria have also shown activity against susceptible and resistant strains of wide range of Gram-positive bacteria [58]. In particular, the human commensal *Staphylococcus lugdunensis* produces lugdunin, which is a thiazolidine-containing cyclic peptide antibiotic that prohibits colonization by *S. aureus* [58]. Lugdunin showed a potent antimicrobial activity against VISA strains (MIC = 3 μg/mL) and did not show lysis of human neutrophils and erythrocytes [58].

On the other hand, a great variety of AMPs derived from bacteria of the *Bacillus* genus with different biological functions have been identified. In particular, the BCP61 peptide produced by bacteria of the *Bacillus* genus was isolated from a fermented food of Asian origin called “kimchi” [113,114]. This AMP has shown activity against different Gram-positive bacteria, such as *S. aureus* and *E. fecalis*. More specifically, it showed potent antibacterial activity against resistant VRSA strains (MIC = 10 μg/mL) [45]. The AMP P138-C—derived from *Bacillus subtilis*, subsp. *inaquosorum*, strain KCTC 13429 and present in a fermented food product—showed activity against a wide diversity of Gram-positive bacteria [39]. This peptide showed MIC of 20 μg/mL and MBC of 640 μg/mL against VRSA strains, and its activity was enhanced when combined with antibiotics, such as oxacillin, ampicillin, and penicillin [39]. Additionally, the peptide CSPK14 derived from *Bacillus amyloliquefaciens* showed activity against VRSA strains with an MIC of 64 μg/mL [38]. The effect of this peptide against these strains was enhanced when tested in synergy with the antibiotics ciprofloxacin and ampicillin [38]. On the other hand, from bacteria of the *Paenibacillus* genus, some naturally occurring peptides with antimicrobial potential have been identified [57]. In particular, fusaricidins (LI-F) are a family of cyclic lipodepsipeptide with antimicrobial activity against a variety of fungi and Gram-positive bacteria [57]. A total of 18 fusaricidin A analogs were designed and synthesized, and then evaluated against ATCC strains of *S. aureus* [57]. In this respect, the analogs 5, 6, 8, 11, and 14 showed the lowest MIC values against VISA strain Mu50 (MIC = 16 μg/mL) and considerable hemolysis [57]. 

#### 3.1.3. Artificial AMPs

Testing of artificial AMPs and their ability to control pathogenic bacteria has gained momentum in recent years because they offer numerous comparative advantages over many natural peptides [115]. For example, many artificial peptides have an enhanced antibacterial effect and fewer adverse effects [80,116]. Thus, de novo design of more stable and effective artificial AMPs and their evaluation is a strategy against infections caused by resistant bacteria, which could be of great clinical importance. Artificial AMPs that demonstrated antimicrobial activity against VRSA and VISA strains are summarized in Table 4. An example of the application of de novo peptide design with activity against Gram-positive bacteria is the LTX-109 peptide designed by Lytix Biopharma [117]. This AMP is emerging as a topical therapeutic alternative against diabetic foot bacterial infections caused by *S. aureus*, as it has shown to be highly effective against resistant clinical isolates [36,117]. This AMP in particular has shown a strong bactericidal effect against VISA and VRSA clinical isolates (MIC = 2–4 μg/mL), demonstrating that the LTX-109 peptide has an antibacterial effect regardless of the resistance patterns of the strains [36,117] (Table 4). Omiganan, an analog peptide of indolicidin, has demonstrated broad-spectrum activity against Gram-positive and Gram-negative bacteria and fungi [118]. This AMP has shown strong activity against VRSA strains, showing a MIC of 16 μg/mL against VRSA and VISA strains [42]. In this regard, omiganan is emerging as a topical treatment used primarily against catheter-related local and bloodstream infections caused by resistant *S. aureus* strains [42,119]. In addition to de novo design, many researchers are using other strategies to enhance the antimicrobial activity and decrease the hemolytic or cytotoxic effects of AMPs [80,116]. Among the strategies that have shown promising results in the design of artificial AMPs we can highlight the following: addition of amino acids to AMPs canonical sequences, synthesis of hybrid peptides by combining sections of different peptides, synthesis of shorter peptides derived from canonical sequences of longer AMPs or proteins, and rational substitution of amino acids in the canonical sequences of AMPs [80,116]. With these strategies, it is possible to manage and modify physicochemical properties of AMPs, such as net charge, hydrophobicity, and amphipathicity [80,116]. In this regard, AMPs, such as MP196, WR12, and DIK-8, designed exclusively with highly specific amino acids, showed antibacterial activity against *S. aureus* strains resistant to conventional antibiotics [37]. The hexapeptide MP196 is a short sequence rich in tryptophan (W) and arginine (R) residues with chemical modifications, such as organoleptic derivatization, fatty acyl, and multivalent studies with promising antimicrobial characteristics [51]. This peptide showed antibacterial activity against VISA strains, with MIC between 16 and 64 μg/mL, and had no significant hemolytic or cytotoxic effects when evaluated against erythrocytes, rat kidney epithelial cells, and human T-cell lymphoblasts [51]. Likewise, the WR12 peptide, also composed exclusively of W and R residues, exhibited broad-spectrum antimicrobial activity, showing very strong activity against VRSA and VISA strains (MIC = 1–8 μM) [37]. DIK-8 is a short AMP composed exclusively of the amino acids isoleucine (I) and lysine (K), which showed antibacterial activity against VRSA (MIC = 8–16 μM) and VISA (MIC = 8 μM) strains, and low toxicity against mammalian cells [37]. Additionally, the design of AMPs by substituting and adding special amino acids has been used to improve antimicrobial activity and reduce the detrimental impact on host cells [120]. For example, the peptide P-113 derived from the human salivary protein histatin 5, which showed antibacterial activity against VRSA and VISA strains (MIC > 64 μg/mL), had its histidine (H) residues replaced by bulky unnatural amino acids [43]. This way the Phe-P-113, Bip-P-113, Dip-P-113, and Nal-P-113 peptides were obtained, which showed an enhanced antibacterial effect against VRSA and VISA strains (Table 4). On the other hand, AMPs with added lipoamino acids have been designed, namely, lipopeptides (lipopeptide-1 to -6). These molecules have shown broad antimicrobial activity against Gram-positive bacteria, including VRSA and VISA strains. However, they have shown toxicity against embryonic and renal cells [40,43] (Table 4). Additionally, other family of artificial small lipopeptides was designed and constructed with a combination of two or three basic, cationic, and/or anionic amino acids attached to an acyl chain of 14 carbons [59]. Seven peptides of this family (C14-KK, C14-RRR, C14-LK, C14-RW, C14-WR, C14-KWI, and C14-LKK) showed antibacterial activity against VISA strain Mu50 with MIC values between 1.56 and >12.5 μM, and strong hemolytic activity against human red blood cells [59]. Finally, two short artificial peptides (RRIKA and RR) exhibited potent and rapid antimicrobial effect against VRSA and VISA clinical isolates with MIC between 2 and 32 μM [47]

### 3.2. AMPs Classification Based on Their Physicochemical and Structural Properties

Antimicrobial peptides have various physicochemical and structural properties that play a key role in regulating their antimicrobial activity, their mechanism of action, and their specificity towards molecular targets [121,122,123,124]. In this sense, AMPs with antibacterial activity against VRSA and VISA strains have different physicochemical properties in terms of amino acid sequence, charge, hydrophobicity, and isoelectric point, which determine their activity against these resistant strains (Table 5, Table 6 and Table 7). Likewise, these peptides have different structures, which allow them to be grouped into four categories: α-helical peptides, β-pleated sheet peptides, mixed-structure peptides (α-helix and β-pleated sheet) (Table 5), and peptides with atypical structure, which include cyclic and complex AMPs, as well as AMPs with unusual amino acids (Table 6 and Table 7). The physicochemical structures and properties of some peptides were reported in some of the papers included in this review. However, when a paper did not report these characteristics for any AMP, its respective prediction was made from the amino acid sequences using the servers I-TASSER (https://zhanglab.ccmb.med.umich.edu/I-TASSER/, accessed on 1 June 2021), ThermoFisher (https://www.thermofisher.com/co/en/home/life-science/protein-biology/peptides-proteins/custom-peptide-synthesis-services/peptide-analyzing-tool.html, accessed on 1 June 2021), and CALCAMPI (https://ciencias.medellin.unal.edu.co/gruposdeinvestigacion/prospeccionydisenobiomoleculas/InverPep/public/herramientas, accessed on 1 June 2021).

#### 3.2.1. α-helix AMPs

AMPs with α-helix structure are the most widely spread in nature [125]. This conformation is important for interaction with bacterial membranes, especially because of the arrangement of amino acids in the helix conformation, where polar residues are segregated on the polar side of the helix and hydrophobic residues on the apolar side of the helix [125,126]. This results in the production of an amphipathic α-helical structure that provides the ability to insert into the hydrophobic sector of bacterial lipid bilayers and cause lethal damage [125,126]. In general, AMPs that adopt this conformation tend to be short and easy to synthesize, as well as having a wide range of antibacterial mechanisms of action [127]. A total of 26 AMPs with antibacterial activity against VRSA and VISA form α-helical structure, with net charges ranging from −2.9 to +9 (Table 5). Cecropins are characterized by their tendency to form α-helical structures [88]. For example, cecropin A, composed of 37 amino acids in length, charge of +6, and an isoelectric point of 10.6, exhibits an α-helical pattern with hydrophobic charged surfaces that make it a highly amphipathic AMP [128]. Likewise, other insect-derived AMPs, such as agelaia-MPI, protonectin, and protonectin-F, also exhibit a α-helical structure, charge of +1, and high incidence of hydrophobic amino acids [48]; a conserved glycine (G) residue gives the flexibility to all these peptides [48]. In contrast, both cathelicidin LL-37 and the derived peptides LL-13 and LL-17 form α-helical structure (Table 5). LL-37 is an AMP consisting of 37 amino acids, 34.6% of which are hydrophobic residues, has a net charge of +6, and has an isoelectric point of 11.1 [129] (Table 5). AMPs synthesized by *A. mellifera* with activity against VRSA and VISA strains, such as melittin and Hec, also form α-helical structure (Table 5). Melittin is an AMP that is synthesized in the bee venom gland as a 70-amino acid propeptide, which is subsequently cleaved to its compact form consisting of 26 residues [130]. The first 20 amino acids of this AMP have polar properties, while the remaining six are hydrophobic, and therefore their net charge of +6 at physiological pH is distributed as +4 in the *N*-terminal region and +2 in the *C*-terminal region [130,131] (Table 5). The Hec peptide is characterized by a high incidence of positively charged amino acids, an α-helix structure, high cationic charge (+9), and a high percentage of hydrophobic amino acids [35]. Peptides, such as smp24 and ctriporin, synthesized by different scorpion species, are structurally composed of a single α-helix (Table 5). The AMP smp24, synthesized by the scorpion *S. maurus*, is composed of 24 amino acids and has a +4 net charge, high hydrophobicity, and a helical structure extending from the *N*-terminal residue to residue 18 [54] (Table 5). This peptide features an alteration in the central proline residue to enhance antibacterial activity; specifically, a kink in the middle of the α-helix structure provides the AMP potent pore formation and selective antimicrobial activity by prokaryotic membranes [54]. Ctriporin forms an α-helix structure, comprising mainly a hydrophobic face and a hydrophilic face [29]. This AMP, with a net charge of +2 due to two positively charged lysine residues, contains more than 50% of hydrophobic amino acids [29]. Magainins produced by amphibians are a family of AMPs that has been structurally well characterized, and many of its members form α-helical structures [132]. One of them is magainin-2, which has a net charge of +3 and is composed mainly of L-amino acids forming a sequence of 23 residues, 43% of which are hydrophobic [132]. Temporins, composed of 13 and 14 amino acids, exhibit an α-helical pattern and net charges ranging from +1 to +2. All temporins with antibacterial activity against VISA strains have more than 50% of hydrophobic residues [56]. Additionally, fallaxin analogs exhibit a α-helical structure; these peptides consisting of 25 amino acids, with more than 50% of hydrophobic residues, have a net charge between +2 and +3 [60]. Several bacteriocins form α-helix structure, such as AMP CSPK-14, which has a low molecular weight (10 kDa), a net charge of −2.9, and no hydrophobic amino acids [38,133]. The antibacterial activity of AMPs with anionic charges are enhanced due to the presence of divalent cations, such as Ca^+2^, Mg^+2^, and Mn^+2^, which allow for the formation of an ion–peptide complex that reduces the overall negative charge of the AMP and favors the affinity for bacterial membranes [134]. In this regard, CSPK-14 improved its bactericidal activity when synergistically evaluated with metal ions, such as Ca^+2^ [38]. Finally, three artificial AMPs form amphipathic α-helix: WR12, RR, and RRIKA [37,47]. WR12, composed of 12 very particular amino acids, contains six arginines and six tryptophans, and therefore it has 50% hydrophobicity and a net charge of +6 helix [37]. RR, composed of 11 amino acids, 54% of which are hydrophobic amino acids, has a net charge of +5, while RRIKA, with 14 amino acids and 57% of hydrophobicity, has a net charge of +6 [47] (Table 5).

#### 3.2.2. AMPs Forming β-Pleated Sheet Peptides

Natural peptides with β-folded sheet conformation are quite widespread in nature and are characterized by their potent antimicrobial activity and their important role in the immune system as immune response regulators [121]. Additionally, AMPs with this type of conformation have been designed to improve their applications and have been widely studied due to the relationship between their structure and the bioactive functions they perform in cells [135]. Peptides with β-folded sheet structure have demonstrated to have antimicrobial activity and selectivity similar to peptides with α-helix structure, according to their physicochemical properties, such as hydrophobicity and net charge [136]. Despite this, it has been found that using short sequences and repeated amino acid segments has been an effective strategy to improve the broad-spectrum activity and selectivity of AMPs with these structures [137]. In this regard, among the AMPs that showed activity against VRSA and VISA strains, DIK-8 is the only one with a secondary structure composed exclusively of a β-folded sheet, as reported by the authors [37]. This peptide has a length of eight amino acids, made up of two sets of a repeated sequence of four amino acids: IRIKIRIK [37]. As for its physicochemical properties, this AMP has a charge of +4, a hydrophobic amino acid content of more than 50%, and an isoelectric point of 12.5 [37].

#### 3.2.3. Mixed AMPs

Mixed AMPs are those that structurally form α-helix combined with folded β-sheet. The AMPs contained in this structure occur naturally and are widespread in nature, showing diverse physicochemical properties and different mechanisms of action [138,139]. Five AMPs with antibacterial activity against VRSA and VISA combine α-helix and β-folded sheet structures, with net charges ranging from +1.9 to +6 (Table 5). In this regard, different members of defensins are characterized by preserving α-helix and β-folded sheet structural motifs, which are stabilized through disulfide bridges [140]. These motifs are highly preserved and have been observed in some species of insects, mussels, plants, and fungi [140]. Formin C is a defensin synthesized by the common housefly *M. domestica*, composed of 40 amino acids, 40% of which are hydrophobic amino acids [46]. This AMP has a net charge of +3, an isoelectric point of 8.3, and a structure composed of an α-helix and two anti-parallel β-folded sheets [46] (Table 5). Likewise, tick-derived AMPs, such as IP, IR, HAE, and OMBAC, are defensins whose structure is also composed of an α-helix and two anti-parallel β-folded sheets, similar to formicin C (Table 5). Despite this, these AMPs possess different physicochemical properties, in terms of net charge at physiological pH, isoelectric point, hydrophobicity, and percentage of hydrophobic residues (Table 5).

#### 3.2.4. AMPs of Atypical Structure: Cyclic, Complex, and with Unusual Amino Acids

Another classification that includes AMPs with structural characteristics different from the conventional ones is peptides that have unusual amino acids or cyclic structures (Table 6 and Table 7). In general, these AMPs are cationic, with 9 to 60 residues and low molecular weight. They have different action mechanisms, which are mainly based on the permeabilization of the bacterial cell membrane [108]. This category includes peptides, such as bacteriocins, which are characterized by cyclic structures of polypeptide chains, where the amino acid residues are covalently linked to form a ring that is favored by the interaction between chemical bonds, such as amide, lactone, ether, thioether, or disulfide [141]. Lantibiotic bacteriocins of subclass Ia (such as nisin, hominicin, and mutancin 1140) and of subclass Ib (such as mersacidin), are small AMPs with molecular weights less than 5 kDa possessing between 19 and 38 amino acids with post-translational modifications [108]. Subclass Ia AMPs are elongated peptides with positive charges, whereas those of subclass Ib are globular and rigid with negative charges [108]. Nisin is an AMP containing 34 amino acids with five rings based on lanthionine or methyllanthionine from the N-terminal to the C-terminal end [142] (Table 6). This peptide is formed from the post-translational modification of an inactive 21 amino acid precursor synthesized by the precursors NisinA, NisinB, and NisinC, which catalyze the dehydration of serine and threonine residues and participate in the cyclization of cysteine [143]. Mutancin 1140 is characterized by having four thioether rings in its chemical structure and a molecular weight of 2.26 kDa, while mersacidin consists of 20 amino acids and forms four intramolecular thioether bridges that form a compact globular structure. It is characterized by a net charge of −1.2, an isoelectric point of 3.3, and a high percentage of hydrophobic amino acids [55,144] (Table 6). Homicin is a bacteriocin with a molecular weight of 2.03 kDa that does not have a specific tertiary structure and possesses thermotolerant properties and high stability [52]. In contrast, bactophencin A, a non-antibiotic bacteriocin, is a cationic AMP consisting of 22 amino acids linked in a loop through a disulfide bond between cysteine residue 7 and 22 [145]. Especially in analog 5, the methionine residues of the original peptide were replaced by the amino acid leucine at positions 14 and 18, and therefore its physicochemical properties show that it is an AMP with a net charge of +7 at physiological pH and with a hydrophobicity of 27% [28]. AMP BCP61 is another bacterial peptide with an atypical structure consisting of nine amino acids, a low percentage of which are hydrophobic, and which has a net charge equal to −1 and an isoelectric point of 3.1 [45]. Lugdunin is a small cyclic bacterial peptide of 0.78 kDa, comprising an unusual thiazolidine heterocycle and five amino acids [58]. Finally, fusaricidin analogs were prepared through modification of the lipid tail, substitution of amino acid, and ester-to-amide substitution [57]. In this respect, LI-F04a analogs 5, 6, 8, and 11 comprising a lipid tail of 12-guanidinododecanoic acid and macrocyclic ring consisting of six amino acids, four of which, Thr1, D-Val2, D-Asn5, and D-Ala6, are conserved throughout all peptides [58] (Table 6). 

Some artificial peptides also present, in their sequences, unusual amino acids and atypical structures, which may provide them with some advantages over natural AMPs [80] (Table 7). These AMPs are mainly characterized by their lower molecular weights. In this sense, omiganan is an artificial cationic peptide with a charge of +5 and an amphipathic nature, and 50% of its amino acids are hydrophobic [42]. The secondary structure of omiganan is very similar to that of its canonical analog indolicidin, as it preserves many of its core elements [146]. In particular, indolicidin presents a disordered structure in aqueous environments, but in the presence of lipid bilayers this AMP adopts a unique and flexible poly-l-proline type II helix structure [146]. The MP196 peptide is a W- and R-rich hexapeptide with a net charge of +2 and an isoelectric point of 12.8. However, the structural conformation of this AMP has not yet been reported [51]. Additionally, peptides that possess non-natural amino acids can also form atypical structural folding. In this regard, from the sequence of peptide P-113, substitutions of H residues by natural amino acids, such as phenylalanine (F) (Phe-P-113), and bulky unnatural ones, such as β-(4,4′-biphenyl)alanines (Bip) (Bip-P-113), β-diphenylalanine (Dip) (Dip-P-113), and β-naphthylalanine (Nal) (Nal-P-113), were performed [147] (Table 2). P-113 (AKRHHGYKRKFH) is an AMP with a net charge of +5, an isoelectric point of 11.6, and 16.7% hydrophobic residues. However, it does not form a typical structure based on α-helix or β-folded sheet [147]. The peptide Phe-P-113 (AKRFFGYKRKFF) has a molecular weight of 1.6 kDa, a net charge of +5, and a hydrophobicity of 32.4. Although these AMPs possess natural amino acids in their sequence, none formed a typical structure based on α-helix or β-folded sheet. Bip-P-113 (AKRBipBipGYKRKKFBip) has a molecular weight of 1.9 kDa, and showed salt resistance, proteolytic stability, and enhanced permeabilization [43]. Nal-P-113 (AKRNalNalGYKRKFNal) has a molecular weight of 1.79 kDa, while Dip-P-113 (AKRDipDipGYKRKKFDip) has a molecular weight of 1.9 kDa [43]. None of these AMPs with unnatural amino acids in their sequence possess a typical structure based on α-helix or β-folded sheet (Table 7). Finally, lipoamino acids were added to lipopeptides −1 to −6 (Table 7). These AMPs possess atypical structural features, as they were constructed according to cyclic and linear configurations with variations in the number of the K residue and lipoamino acids with 12 carbon atoms [40]. These peptides are cationic, possess less than eight amino acids, and have different grades of hydrophobicity [40].

**Table 6:** AMPs of atypical structure derived from bacteria that showed antibacterial activity against VRSA and VISA strains.

AMP Name	Aminoacid Sequences and Structures	Molecular Weight (KDa)	Reference
Nisin	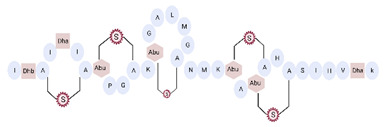	3.35	[53]
Hominicin	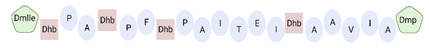	2.03	[52]
Mutacin 1140 (MU1140)	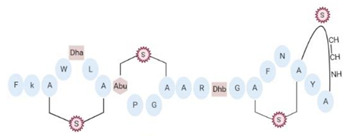	2.26	[44]
Mersacidin	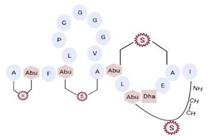	1.82	[55,127]
Bactofencin A (analog 5)	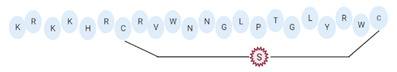	2.77	[28]
BCP61	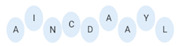	9.50	[45]
Lugdunin	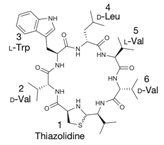	0.78	[58]
LI-F04a analog 5	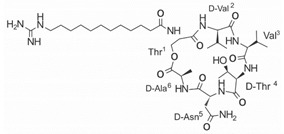	–	[58]
LI-F04a analog 6	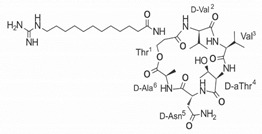	–	[58]
LI-F04a analog 8	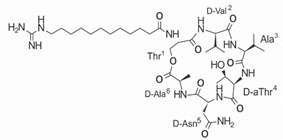	–	[58]
LI-F04a analog 11	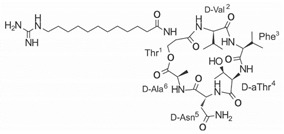	–	[58]

Abbreviations: Dha, dehydroalanine; Dhb, dehydrobutyrine; Abu, aminobutyrine; DmIle, dimethyl-isoleucine; Dmp, propanediamine. Dashes indicate information was not determined or was not included in the reference.

**Table 7:** Artificial AMPs of atypical structure that showed antibacterial activity against VRSA and VISA strains.

Peptide Name	Chemical Structure	Molecular Weight (KDa)	Reference
Omiganan	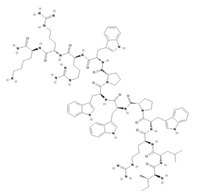	1.96	[42,148]
Lipopeptide 1 *	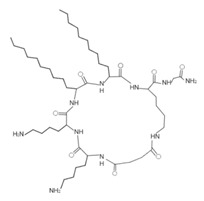	–	[40]
Lipopeptide 2 *	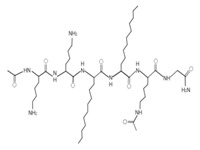	–	[40]
Lipopeptide 3 *	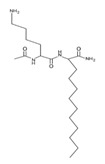	–	[40]
Lipopeptide 4 *	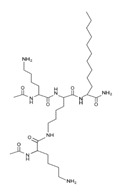	–	[40]
Lipopeptide 5 *	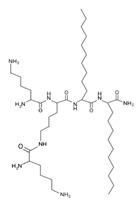	–	[40]
Lipopeptide 6 *	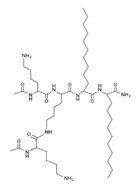	–	[40]

* Stereochemistry was not reported for these AMPs. Dashes indicate information was not determined or was not included in the reference.

### 3.3. Mechanisms of Action of AMPs with Antibacterial Activity against VRSA and VISA

**Figure 1:**
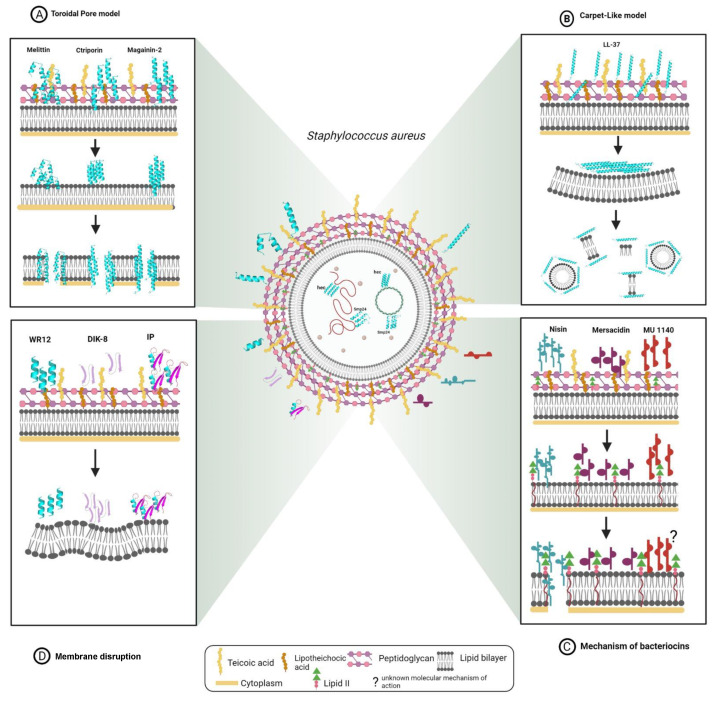
Mechanisms of action model of AMPs that showed antibacterial activity against VRSA and VISA strains. (**A**) AMPs that cause the formation of pores and cracks in the bacterial membrane; (**B**) AMPs that interact with the bacterial membrane causing its disruption through carpet model; (**C**) AMPs that cause a bactericidal effect through disruption and damage of the membrane; (**D**) Bacteria-derived AMPs that that interact with the bacterial membrane causing disruption and the formation of pores.

The action mechanisms of various AMPs are not yet fully defined, but several studies have been conducted to determine how the peptides can kill bacteria, and therefore this is an important area of study that is beginning to gain significant importance [149]. The action mechanism of peptides with antimicrobial activity is determined by and largely depends on their physicochemical properties and the molecular target they act upon [149]. Different molecular mechanisms have been described for antibacterial AMPs, including interaction with the membrane to induce damage; interaction with DNA, RNA, and proteins to inhibit their activity; or interaction with molecules at the cytoplasmic level to inhibit the synthesis of proteins, enzymes, and nucleic acids, as well as affect protein folding [26,27,150]. The interaction of AMPs with the bacterial wall depends on the composition of the cell wall and the nature of the phospholipid composition [150]. The initial step for the interaction of AMPs with bacteria is mediated by electrostatic attraction between the anionic components present in the bacterial membrane and the peptides [149]. Once peptides enter the bacterial cell, they can destroy bacteria by interfering with some major pathways crucial for survival, such as cell wall synthesis, protein synthesis, and DNA replication [149]. In Gram-positive bacteria, AMPs first interact with the teichoic and lipoteichoic acid present in the cell wall, then insert themselves into the thick peptidoglycan layer, and finally encounter the cytoplasmic membrane containing negatively charged phospholipids [151]. This is where the physicochemical properties of AMPs, such as hydrophobicity, improve their antibacterial performance against resistant strains of *S. aureus*, since they facilitate interaction with the bacterial lipid bilayer [152]. However, AMPs with very high hydrophobicity values have also been observed to have negative effects, such as increased cytotoxicity in mammalian cells, loss of selectivity, self-association tendencies, and aggregation [153]. AMPs with antibacterial activity against VRSA and VISA strains show diverse molecular mechanisms of action. Thus, they can be grouped according to their molecular targets as peptides that permeabilize the membrane and peptides that interact with intracellular components (Figure 1).

#### 3.3.1. AMPs That Permeabilize Bacterial Membranes

The most widespread action mechanism among antibacterial peptides is the capacity of AMPs to alter bacterial membranes [121]. AMPs can act on specific membrane components or, on the contrary, cause a generalized disruption of the bacterial lipid bilayer [24]. In particular, anionic lipids present in the membranes of Gram-positive bacteria, such as phosphatidylglycerol and cardiolipin, attract AMPs favoring selectivity for bacterial membranes [121,154]. Peptides have demonstrated high capacity to penetrate the cell wall, causing severe damage leading to bacterial death, especially against *S. aureus*. However, resistant strains may represent a new challenge for AMPs [76]. In this context, knowing the particular structure of the membranes of these bacterial strains allows us to establish the specific mechanism of action used by each AMP to induce bacterial death [24]. The peptides with activity against VRSA and VISA strains are characterized by being highly potent and fast-acting AMPs. However, the specific mechanism of action for most of them is unknown. Despite this fact, it has been described that some AMPs interact with the membranes of these strains causing their disruption through two mechanisms of action: toroidal pore model and carpet model [24]. In the toroidal pore model, the AMPs are embedded vertically and accumulate in the cell membrane forming a hole with a diameter of 1 to 2 nm, through the interaction of peptides with the phospholipid head groups [24]. Melittin and ctriporin are examples of AMPs that formed toroid pores in the membranes of VRSA and VISA strains [29,155]. These AMPs cause the formation of pores and cracks in the cell membrane, leading to leakage of cytoplasmic content and causing bacterial death [29,155] (Figure 1A). In the carpet model, AMPs are arranged parallel to the cell membrane, with the hydrophilic ends facing the solution and the hydrophobic ends facing the phospholipid bilayer [24]. Thus, AMPs coat the bacterial membrane surface in a carpet-like manner, creating unfavorable alterations that lead to the disintegration and destruction of the lipid bilayer in a detergent-like manner [24,121]. Human cathelicidin LL-37 exhibits this mechanism of action, causing disruption of the bacterial membrane and creating channels or pores leading to cell death [129] (Figure 1B). Magainin-2 has mainly shown the toroidal pore mechanism against *S. aureus*. However, it was shown that when this peptide was incorporated into liposomes comprising phosphatidylserine or phosphatidylethanolamine, it exhibited a detergent-like mechanism of action, comparable to the carpet model [156] (Figure 1A). On the other hand, some peptides that showed activity against VRSA and VISA strains, although they did not exhibit mechanisms, such as toroid pore and carpet, interacted with the bacterial membrane of *S. aureus*, leading to its disruption [28,34,37,51,88] (Figure 1). In this sense, IP peptides [34], bactophencin A [28], lipopeptide-2 [40], cecropin A, WR12, DIK-8, and MP196 achieve membrane permeabilization, causing a bactericidal effect through disruption and damage of the membrane of *S. aureus* strains [37,51,88] (Figure 1C). Finally, bacteriocins, such as nisin, mutancin 1140, and mersacidin, specifically target cell wall components, such as lipid II, by interfering with peptidoglycan synthesis and can also be inserted into the membrane causing pores that lead to leakage of intracellular components [44,55,143] (Figure 1D).

#### 3.3.2. AMPs That Interact with DNA

Once the AMPs have entered the bacterial cell, they identify and act on their specific molecular target [24]. Depending on the molecular target, AMPs can inhibit protein or nucleic acid synthesis, or affect different metabolic and cell cycle activities [24]. In particular, some AMPs can affect key enzymes or induce the degradation of nucleic acid molecules to inhibit nucleic acid biosynthesis [24]. Some antimicrobial peptides that showed activity against VRSA and VISA strains showed interaction with DNA (Figure 1). Gel electrophoresis assays showed that the Hec peptide causes DNA damage, and that smp24 causes membrane permeabilization and subsequently binds to DNA [35,54]. Finally, the action mechanism of omiganan is similar to that of indolicidin. This AMP translocates to the cytoplasm and inhibits DNA replication by affecting thymine incorporation [157].

## 4. Conclusions

AMPs represent a new hope as an alternative in the control of resistant strains of *S. aureus*. Several antimicrobial peptides naturally produced by different species of animals and bacteria have shown in vitro and in vivo antibacterial activity against VRSA and VISA strains and clinical isolates with multiple-drug resistance. Due to the diversity in their physicochemical and structural properties, AMPs can kill bacteria by different mechanisms and have shown little likelihood of inducing resistance, and therefore they have great comparative advantages over conventional antibiotics in the control of VRSA and VISA strains and clinical isolates. However, some peptides may possess hemolytic or cytotoxic effects. In order to reduce adverse effects and enhance antibacterial activity, several methods have been implemented to design artificial AMPs, which have shown great therapeutic potential for controlling resistant strains. Despite this, the specific action mechanism deployed by most AMPs to kill these resistant strains is not fully understood. For this reason, it is necessary to study the molecular action mechanism of AMPs through genotypic and phenotypic characterization of the strains and implementation of different computational and laboratory methods, such as membrane simulation and molecular dynamics. Moreover, this will provide relevant information to study resistance to AMPs. We believe that advances in the discovery, design, optimization, synthesis, and evaluation of both natural and artificial antimicrobial peptides is an excellent way to develop potential alternatives for the control of resistant bacteria. Use of AMPs with potent and rapid antibacterial activity and non-toxicity to human cells will have a strong impact in the future of clinical practices for treatment of infectious diseases. However, the discovery and development of this kind of AMPs, as well the possible resistance of bacteria to AMPs will be the next challenges.

## Figures and Tables

**Table 1:** Resistance profile of VRSA and VISA strains that showed susceptibility to AMPs.

Strain ID *	Interpretive Categories for Conventional Antibiotics									Method	Genotype	Reference
	PEN ^1^	AMX ^1^	OXA ^1^	ERY ^2^	VAN ^3^	TET ^4^	DAP ^5^	LZD ^6^	CLI ^7^			
VRSA-1	R	–	–	–	R	–	–	–	–	MIC (μg/mL)	–	[29]
VRSA-2	R	–	–	–	R	–	–	–	–	MIC (μg/mL)	–	[29]
VRSA-3	–	–	–	–	R	–	–	–	–	–	–	[34]
VRSA-4	R	R	–	R	R	S	–	–	–	Disc diffusion	*VanA*	[35]
VRSA-5	–	–	–	–	R	–	S	S	R	MIC (μg/mL)	SCCmec II	[36]
VRSA-6	–	–	R	–	R	–	–	–	–	MIC (μM)	–	[37]
VRSA-7	–	–	R	–	R	–	–	–	–	MIC (μM)	–	[37]
VRSA-8	–	–	R	–	R	–	–	–	–	MIC (μM)	–	[37]
VRSA-9	–	–	R	–	R	–	–	–	–	MIC (μM)	–	[37]
VRSA-10	–	–	R	–	R	–	–	–	–	MIC (μM)	–	[37]
VRSA-11	–	–	R	–	R	–	–	–	–	MIC (μM)	–	[37]
VRSA-12	–	–	R	–	R	–	–	–	–	MIC (μM)	–	[37]
VRSA-13	R	–	R	–	R	–	–	–	–	MIC (μg/mL)	–	[38]
VRSA-14	–	–	–	–	R	–	–	–	–	MIC (μg/mL)	–	[39]
VRSA-15	–	–	–	–	R	–	–	–	–	MIC (μM)	–	[40]
VRSA-16	–	–	–	–	R	–	–	–	–	MIC (μM)	–	[40]
VRSA-17	–	–	–	–	R	–	–	–	–	MIC (μM)	–	[40]
VRSA-18	–	–	–	–	R	–	–	–	–	MIC (μg/mL)	–	[41]
VRSA-19	–	–	S	R	R	R	–	–	R	MIC (μg/mL)	–	[42]
VRSA-20	–	–	–	-	R	–	–	–	–	MIC (μg/mL)	–	[43]
VRSA-21	–	–	–	-	R	–	–	–	–	MIC (μg/mL)	–	[43]
VRSA-22	–	–	–	-	R	–	–	–	–	MIC (μg/mL)	–	[43]
VRSA-23	–	–	–	-	R	–	–	–	–	MIC (μg/mL)	–	[44]
VRSA-24	–	–	–	-	R	–	–	–	–	MIC (μg/mL)	–	[45]
VRSA-25	–	–	–	-	R	–	–	–	–	–	–	[28]
VRSA-26	–	–	–	-	R	–	–	–	–	–	–	[28]
VRSA-27	–	–	–	-	R	–	–	–	–	MIC (μg/mL)	*MecA*	[46]
VRSA-28	–	–	–	-	R	–	–	S	–	–	–	[47]
VRSA-29	–	–	–	-	R	–	–	S	–	–	–	[47]
VRSA-30	–	–	–	-	R	–	–	S	–	–	–	[47]
VRSA-31	–	–	–	-	R	–	–	S	–	–	–	[47]
VRSA-32	–	–	–	-	R	–	–	R	–	–	–	[47]
VRSA-33	–	–	–	-	R	–	–	–	–	–	–	[48]
VISA-1	R	–	–	-	I	–	–	–	–	MIC (μg/mL)	–	[29]
VISA-2	–	–	–	-	I	–	–	–	–	MIC (μg/mL)	–	[34]
VISA-3	–	–	–	-	I	–	R	S	R	MIC (μg/mL)	SCCmec II	[36]
VISA-4	–	–	–	R	I	–	–	S	–	MIC (μM)	–	[37]
VISA-5	–	–	–	R	I	–	–	S	–	MIC (μM)	–	[37]
VISA-6	–	–	–	R	I	–	–	S	–	MIC (μM)	–	[37]
VISA-7	–	–	–	–	I	–	–	–	–	MIC (μg/mL)	–	[41]
VISA-8	–	–	–	–	I	–	–	–	–	Disc diffusion	–	[49]
VISA-9	–	–	–	–	I	–	–	–	–	MIC (μM)	–	[50]
VISA-10	–	–	S	R	I	S	–	–	R	MIC (μg/mL)	–	[42]
VISA-11	–	–	S	R	I	S	–	–	R	MIC (μg/mL)	–	[42]
VISA-12	–	–	–	–	I	–	–	–	–	MIC (μg/mL)	–	[43]
VISA-13	–	–	–	–	I	–	–	–	–	MIC (μg/mL)	–	[43]
VISA-14	–	–	–	–	I	–	–	–	–	MIC (μg/mL)	–	[43]
VISA-15	-	–	–	–	I	–	–	–	–	MIC (μg/mL)	–	[44]
VISA-16	-	–	–	–	I	–	–	–	–	MIC (μg/mL)	*GraS*	[51]
VISA-17	-	–	–	–	I	–	–	–	–	MIC (μg/mL)	*GraS*	[51]
VISA-18	-	–	–	–	I	–	–	–	–	–	–	[52]
VISA-19	-	–	–	–	I	–	–	–	–	–	–	[53]
VISA-20	-	–	–	–	I	–	–	–	–	–	–	[53]
VISA-21	-	–	–	–	I	–	–	–	–	–	–	[53]
VISA-22	-	–	–	–	I	–	–	–	–	–	–	[53]
VISA-23	-	–	–	–	I	–	–	–	–	–	–	[53]
VISA-24	-	–	–	–	I	–	–	–	–	–	–	[53]
VISA-25	-	–	–	–	I	–	–	–	–	MIC (μg/mL)	–	[54]
VISA-26	-	–	–	–	I	–	–	–	–	MIC (μg/mL)	–	[54]
VISA-27	-	–	–	–	I	–	–	–	–	–	–	[55]
VISA-28	–	–	–	–	I	–	–	–	–	–	–	[56]
VISA-29	–	–	–	–	I	–	–	–	–	–	–	[57]
VISA-30	–	–	–	–	I	–	–	–	–	–	–	[58]
VISA-31	–	–	–	–	I	–	–	–	–	–	–	[59]
VISA-32	–	–	–	–	I	–	–	–	–	–	–	[60]
VISA-33	–	–	–	–	I	–	–	S	–	–	–	[47]

Abbreviations: VRSA, vancomycin-resistant Staphylococcus aureus; VISA, vancomycin-intermediate Staphylococcus aureus; MIC, minimal inhibitory concentration; S, susceptible; I, intermediate; R, resistant; PEN, penicillin; AMX, amoxicillin; ERI, erythromycin; VAN, vancomycin; TET, tetracyclines; DAP, daptomycin; LZD, linezolid; CLI, clindamycin. ^1^ Beta-lactams; ^2^ macrolides; ^3^ glycopeptides; ^4^ tetracyclines; ^5^ lipopeptides; ^6^ oxazolidinones; ^7^ lincosamides. Dashes indicate information was not determined or was not included in the reference. * The strain IDs were adjusted for this review, but the respective correspondence with original IDs were included in Supplementary Materials Table S1.

**Table 2:** Animal-derived AMPs with antibacterial activity against VRSA and VISA strains.

Source	AMP Name	Strain ID	MIC Value	Reference	Toxicity/Properties
*Apis mellifera*	Melittin	VISA-9	2 μM	[50]	High toxicity to erythrocytes and other human cells
Mellitin analog	Hec	VRSA-4	80 μM	[35]	Moderate toxic effect at high concentrations
*Musca domestica*	Formicin C	VRSA-27	32 μg/mL	[46]	Non toxic to the intradermal model of the larva *Hermetia illucens*
*Hyalophora cecropia*	Cecropin A	VISA-8	64 μg/mL	[49]	Low cytotoxic effect on human lung carcinoma
*Parachartergus fraternus* and *Agelaia pallipes pallipes*	Agelaia-MPI	VRSA-33	4–8 μg/mL	[48]	Strong hemolytic effect on human erythrocytes
	Protonectin	VRSA-33	16 μg/mL	[48]	Toxic to cancerous and non-cancerous cell lines, but moderated hemolytic effect against human erythrocytes
Agelaia-MPI analog	NeuroVAL	VRSA-33	>128 μg/mL	[48]	Non toxic to human erythrocytes, and cancerous and non-cancerous cells lines.
Protonectin analog	Protonectin-F	VRSA-33	16 μg/mL	[48]	Toxic to cancerous and non-cancerous cell lines, but moderated hemolytic effect against human erythrocytes
*Chaerilus tricostatus*	Ctriporin	VRSA-1	10 μg/mL	[29]	Histological results showed recovery of the skin
		VRSA-2	10 μg/mL	[29]	
		VISA-1	10 μg/mL	[29]	
*Scorpio maurus palmatus*	Smp24	VISA-25	32 μg/mL	[54]	Toxic to sheep erythrocytes
		VISA-26	64 μg/mL	[54]	
*Ixodes persulcatus*	Persulcatusin (IP)	VRSA-3	2 μg/mL	[34]	Non toxic to fibroblasts, colon epithelial cells, and erythrocytes
		VISA-2	8 μg/mL	[34]	
*Ixodes ricinus*	IR	VRSA-3	32 μg/mL	[34]	–
		VISA-2	>32 μg/mL	[34]	–
*Haemaphysalis longicornis*	HAE	VRSA-3	>32 μg/mL	[34]	–
		VISA-2	>32 μg/mL	[34]	–
*Ornithodoros moubata*	OMBAC	VRSA-3	8 μg/mL	[34]	–
		VISA-2	>32 μg/mL	[34]	–
*Xenopus laevis*	Magainin-2	VISA-8	16 μg/mL	[49]	–
*Lithobates capito*	Temporin-CPa	VISA-28	>25 μM	[56]	Hemolysis of human erythrocytes at high concentrations
	Temporin-CPb	VISA-28	12.5 μM	[56]	Hemolysis of human erythrocytes at high concentrations
*Rana grylio*	Temporin-1Ga	VISA-28	6.2 μM	[56]	Strong hemolytic effect on human erythrocytes
*Rana okaloosae*	Temporin-1OLa	VISA-28	3.1 μM	[56]	Strong hemolytic effect on human erythrocytes
*Rana septentrionalis*	Temporin-1 SPa	VISA-28	12.5 μM	[56]	Moderate hemolytic effect on human erythrocytes
*Rana ornativentris*	Temporin-1Oc	VISA-28	1.6 μM	[56]	Strong hemolytic effect on human erythrocytes
Fallaxin analogs	FL10	VISA-32	50 μM	[60]	High hemolytic effect on human erythrocytes
	FL9	VISA-32	50 μM	[60]	Moderate hemolytic effect on human erythrocytes
	FA12	VISA-32	50 μM	[60]	High hemolytic effect on human erythrocytes
	FL14	VISA-32	50 μM	[60]	High hemolytic effect on human erythrocytes
*Homo sapiens*	LL-37	VRSA-18	64 μg/mL	[41]	Low cytotoxic effect
		VISA-7	64 μg/mL	[41]	
Derived from LL-37	LL-13	VRSA-18	512 μg/mL	[41]	–
		VISA-7	1024 μg/mL	[41]	–
Derived from LL-37	LL-17	VRSA-18	512 μg/mL	[41]	–
		VISA-7	1024 μg/mL	[41]	–

Dashes indicate information was not determined or was not included in the reference.

**Table 3:** Bacteria-derived AMPs with antibacterial activity against VRSA and VISA strains.

Source	AMP Name	Strain ID	MIC Value	Reference	Toxicity/Properties
*Lactococcus lactis*	Nisin	VISA-19	4.1 mg/L	[53]	Hemolytic effect on sheep erythrocytes
		VISA-20	8.3 mg/L	[53]	
		VISA-21	4.1 mg/L	[53]	
		VISA-22	8.3 mg/L	[53]	
		VISA-23	8.3 mg/L	[53]	
		VISA-24	4.1 mg/L	[53]	
*Staphylococcus hominis*	Hominicin	VISA-18	3.82 μg/mL	[52]	–
*Streptococcus mutans*	Mutacin 1140	VRSA-23	4–8 μg/mL	[44]	–
		VISA-15	4 μg/mL	[44]	–
*Bacillus* sp.	Mersacidin	VISA-27	35 μg/mL	[55]	–
*Lactobacillus salivarius*	Bactofencin A (analog 5)	VRSA-25	4.3 μM	[28]	–
		VRSA-26	100 μM	[28]	–
*Staphylococcus lugdunensis*	Lugdunin	VISA-30	3 μg/mL	[58]	No lysis of primary human erythrocytes or neutrophils.
*Bacillus* sp.	BCP61	VRSA-24	10 μg/mL	[45]	–
*Bacillus subtilis subsp. inaquosorum*	P138-C	VRSA-14	20 μg/mL	[39]	–
*Bacillus amyloliquefaciens*	CSPK14	VRSA-13	64 μg/mL	[38]	–
Fusaricidin analogs	LI-F04a analog 5	VISA-29	16 μg/mL	[57]	Hemolysis on human erythrocytes
	LI-F04a analog 6	VISA-29	16 μg/mL	[57]	Hemolysis on human erythrocytes
	LI-F04a analog 8	VISA-29	16 μg/mL	[57]	Hemolysis on human erythrocytes
	LI-F04a analog 11	VISA-29	16 μg/mL	[57]	Hemolysis on human erythrocytes

Dashes indicate information was not determined or was not included in the reference.

**Table 4:** Artificial AMPs with antibacterial activity against VRSA and VISA strain.

AMP Name	Strain ID	MIC Value	Reference	Toxicity/Properties
LTX-109	VRSA-5	2–4 μg/mL	[36]	Phase III of a clinical trial
	VISA-3	2–4 μg/mL	[36]	
Omiganan (Indolicidin analog)	VRSA-19	16 μg/mL	[42]	Topical antimicrobial agent in phase III of a clinical trial
	VISA-10	16 μg/mL	[42]	
	VISA-11	16 μg/mL	[42]	
WR12	VRSA-6	4 μM	[37]	–
	VRSA-7	8 μM	[37]	–
	VRSA-8	8 μM	[37]	–
	VRSA-9	4 μM	[37]	–
	VRSA-10	4 μM	[37]	–
	VRSA-11	8 μM	[37]	–
	VRSA-12	4 μM	[37]	–
	VISA-4	1 μM	[37]	–
	VISA-5	1 μM	[37]	–
	VISA-6	1 μM	[37]	–
DIK-8	VRSA-6	8 μM	[37]	–
	VRSA-7	16 μM	[37]	–
	VRSA-8	16 μM	[37]	–
	VRSA-9	16 μM	[37]	–
	VRSA-10	16 μM	[37]	–
	VRSA-11	16 μM	[37]	–
	VRSA-12	16 μM	[37]	–
	VISA-4	8 μM	[37]	–
	VISA-5	8 μM	[37]	–
	VISA-6	8 μM	[37]	–
MP196	VISA-16	16 μg/mL	[51]	Light hemolytic effect on cell lines of breast cancer. Acute toxicity in mice cells.
	VISA-17	64 μg/mL	[51]	
P-113	VRSA-20	>64 μg/mL	[43]	–
	VRSA-21	>64 μg/mL	[43]	–
	VRSA-22	>64 μg/mL	[43]	–
	VISA-12	>64 μg/mL	[43]	–
	VISA-13	>64 μg/mL	[43]	–
	VISA-14	>64 μg/mL	[43]	–
Phe-P-113	VRSA-20	>64 μg/mL	[43]	–
	VRSA-21	>64 μg/mL	[43]	–
	VRSA-22	>64 μg/mL	[43]	–
	VISA-12	>64 μg/mL	[43]	–
	VISA-13	>64 μg/mL	[43]	–
	VISA-14	>64 μg/mL	[43]	–
Bip-P-113	VRSA-20	16 μg/mL	[43]	–
	VRSA-21	16 μg/mL	[43]	–
	VRSA-22	8 μg/mL	[43]	–
	VISA-12	16 μg/mL	[43]	–
	VISA-13	16 μg/mL	[43]	–
	VISA-14	8 μg/mL	[43]	–
Dip-P-113	VRSA-20	32 μg/mL	[43]	–
	VRSA-21	32 μg/mL	[43]	–
	VRSA-22	32 μg/mL	[43]	–
	VISA-12	16 μg/mL	[43]	–
	VISA-13	16 μg/mL	[43]	–
	VISA-14	16 μg/mL	[43]	–
Nal-P-113	VRSA-20	8 μg/mL	[43]	–
	VRSA-21	8 μg/mL	[43]	–
	VRSA-22	16 μg/mL	[43]	–
	VISA-12	8 μg/mL	[43]	–
	VISA-13	8 μg/mL	[43]	–
	VISA-14	8 μg/mL	[43]	–
Lipopeptide 1	VRSA-15	0.5 μM	[40]	Low toxicity in human embryonic and kidney cells
	VRSA-16	0.7 μM	[40]	
	VRSA-17	0.9 μM	[40]	
Lipopeptide 2	VRSA-15	2.8 μM	[40]	Low toxicity in human embryonic and kidney cells
	VRSA-16	1.9 μM	[40]	
	VRSA-17	2.8 μM	[40]	
Lipopeptide 3	VRSA-15	>30 μM	[40]	Low toxicity in human embryonic and kidney cells
	VRSA-16	>30 μM	[40]	
	VRSA-17	>30 μM	[40]	
Lipopeptide 4	VRSA-15	>30 μM	[40]	Low toxicity in human embryonic and kidney cells
	VRSA-16	>30 μM	[40]	
	VRSA-17	>30 μM	[40]	
Lipopeptide 5	VRSA-15	0.2 μM	[40]	Low toxicity in human embryonic and kidney cells
	VRSA-16	0.1 μM	[40]	
	VRSA-17	0.1 μM	[40]	
Lipopeptide 6	VRSA-15	2.8 μM	[40]	Low toxicity in human embryonic and kidney cells
	VRSA-16	1.9 μM	[40]	
	VRSA-17	1.9 μM	[40]	
C14-KK	VISA-31	12.5 μM	[59]	Strong hemolytic effect on human erythrocytes
C14-RRR	VISA-31	3.1 μM	[59]	Strong hemolytic effect on human erythrocytes
C14-LK	VISA-31	1.56 μM	[59]	Strong hemolytic effect on human erythrocytes
C14-RW	VISA-31	>12.5 μM	[59]	Strong hemolytic effect on human erythrocytes
C14-WR	VISA-31	3.1 μM	[59]	Strong hemolytic effect on human erythrocytes
C14-KWI	VISA-31	12.5 μM	[59]	Strong hemolytic effect on human erythrocytes
C14-LKK	VISA-31	3.1 μM	[59]	Strong hemolytic effect on human erythrocytes
RRIKA	VISA-33	2 μM	[47]	Low hemolytic activity, but show toxicity in mammalian cell lines
	VRSA-29	4 μM	[47]	
	VRSA-30	4 μM	[47]	
	VRSA-31	4 μM	[47]	
	VRSA-32	4 μM	[47]	
	VRSA-33	4 μM	[47]	
RR	VISA-33	16 μM	[47]	Low hemolytic activity, but show toxicity in mammalian cell lines
	VRSA-29	32 μM	[47]	
	VRSA-30	16 μM	[47]	
	VRSA-31	16 μM	[47]	
	VRSA-32	32 μM	[47]	
	VRSA-33	32 μM	[47]	

Dashes indicate information was not determined or was not included in the reference.

**Table 5:** Structural and physicochemical properties of AMPs that showed antibacterial activity against VRSA and VISA strains.

AMP Name	3D Structure	Sequence	L	C	IP	H	%H	Reference
Cecropin A	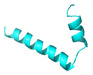	GIGKFLHSAKKFGKAFVGEIMNS	23	+6	10.6	40.19	43.48	[37]
Agelaia-MPI	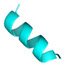	INWLKLGKAIIDAL	14	+1	9.9	45.73	64.29	[48]
Protonectin	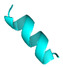	ILGTILGLLKGL	12	+1	10.1	47.67	58.33	[48]
Protonectin-F	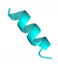	IFGTILGFLKGL	12	+1	10.1	50.16	58.33	[48]
LL-37	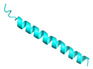	LLGDFFRKSKEKIGKEFKRIVQRIKDFLRNLVPRTES	37	+6	11.1	35.14	34.62	[41]
LL-13	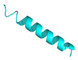	IGKEFKRIVQRIKDFLRNLVPRTES	25	+4	11.4	39.37	36.00	[41]
LL-17	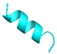	LLGDFFRKSKEKIGKEFKRIVQRIKDFLRNLVPRTES	13	+4	12.2	35.69	46.15	[41]
Melittin	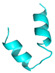	GIGAVLKVLTTGLPALISWIKRKRQQ	26	+5	12.5	49.39	46.15	[50]
Hec	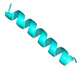	FALALKALKKALKKLKKALKKAL	23	+9	11.4	39.47	60.87	[35]
Smp24	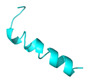	IWSFLIKAATKLLPSLFGGG-KKDS	24	+4	10.6	50.39	45.83	[54]
Ctriporin	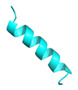	FLWGLIPGAVTSLIAISKK	19	+2	10.6	55.47	57.89	[29]
Magainin-2	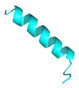	GIGKFLHSAKKFGKAFVGEIMNS	23	+3	10.6	40.19	43.48	[49]
Temporin-CPa	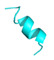	IPPFIKKVLTTVF	13	+2	10.6	41.02	53	[56]
Temporin-CPb	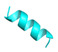	FLPIVGRLISGIL	13	+1	11.1	46.35	61	[56]
Temporin-1Ga	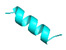	SILPTIVSFLSKVF	14	+1	10.1	52.43	57	[56]
Temporin-1OLa	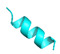	FLPFLKSILGKIL	13	+2	10.6	48.08	61	[56]
Temporin-1Spa	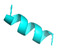	FLSAITSILGKFF	13	+1	10.1	47.05	61	[56]
Temporin-1Oc	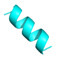	FLPLLASLFSRLF	13	+1	11.1	59.16	69	[56]
FL9	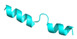	GVVDILKGLAKDIAGHLASKVMNKL	25	+2	10.2	41.54	52	[60]
FL10	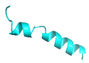	GVVDILKGALKDIAGHLASKVMNKL	25	+2	10.2	41.31	52	[60]
FA-12	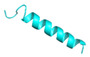	GVVDILKGAAKAIAGHLASKVMNKL	25	+3	10.6	37.87	56	[60]
FL-14	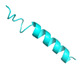	GVVDILKGAAKDILGHLASKVMNKL	25	+2	10.2	41.54	52	[60]
CSPK-14	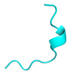	HYDPGDDSGNTG	12	−2.9	3.6	5.66	0	[38]
WR12	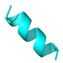	RWWRWWRRWWRR	12	+6	13.2	50.42	50.00	[37]
RR	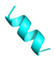	WLRRIKAWLRR	11	+5	13.0	33.04	54	[47]
RRIKA	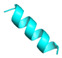	WLRRIKAWLRRIKA	14	+6	13.0	39.90	57	[47]
Formicin C	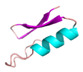	ATCDLLSGTGVGHSACAAHCLLRGNRGGYCNGKGVCVCRN	40	+3	8.3	30.58	42.50	[46]
IP	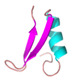	GFGCPFNQGACHRHCRSIGRRGGYCAGLFKQTCTCYSR	38	+6	9.3	29.58	34.21	[34]
IR	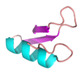	GGYYCPFFQDKCHRHCRSFGRKAGYCGGFLKKTCICV	37	+6	9.2	36.11	37.84	[34]
HAE	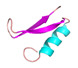	GCPLNQGACHNHCRSIGRRGGYCAGIIKQTCTCYRK	36	+6	9.3	23.43	33.33	[41]
OMBAC	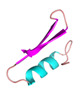	GFGCPFNQYECHAHCSGVPGYKGGYCKGLFKQTCNCY	37	+2	8.0	32.12	32.43	[41]

Abbreviations: L, length; C, net charge; IP, isoelectric point; H, hydrophobicity; %H, percentage of hydrophobicity.

## Supplementary Materials

The following are available online at https://www.mdpi.com/article/10.3390/ijms22157927/s1.
