# Supplementary material for: Antimicrobial Peptides with Antibacterial Activity against Vancomycin-Resistant Staphylococcus aureus Strains: Classification, Structures, and Mechanisms of Action
Source: Int J Mol Sci. 2021 Jul 25;22(15):7927. doi: 10.3390/ijms22157927 (PMC8347216; doi:10.3390/ijms22157927)
Supplement: Supplementary file 1 [file ijms-22-07927-s001.zip › ijms-1278052-supplementary.pdf]

Table S1. Correspondence between strain IDs that were adjusted in the review and the original IDs that were assigned to strains in the reference papers.

Strain IDs that were adjusted in the review	Original IDs assigned to strains in the reference papers	Reference
VRSA-1	P1374	[1]
VRSA-2	P1369	[1]
VRSA -3	VRS1	[2]
VRSA -4	VRSA	[3]
VRSA -5	VRSA AC	[4]
VRSA -6	VRS4	[5]
VRSA -7	VRS5	[5]
VRSA -8	VRS10	[5]
VRSA -9	VRS11a	[5]
VRSA -10	VRS11b	[5]
VRSA-11	VRS12	[5]
VRSA -12	VRS13	[5]
VRSA -13	VRSA	[6]
VRSA -14	VRSA	[7]
VRSA -15	VRS4	[8]
VRSA -16	VRS 1	[8]
VRSA -17	VRS 10	[8]
VRSA -18	BR-VRSA	[9]
VRSA -19	VRSA	[10]
VRSA -20	VRSA01	[11]
VRSA -21	VRSA02	[11]
VRSA -22	VRSA03	[11]
VRSA -23	VRSA	[12]
VRSA -24	VRSA	[13]
VRSA -25	VRSA-40413389	[14]
VRSA -26	VRSA40410425	[14]
VRSA -27	MRSA AC R116	[15]
VRSA-28	VRS1	[16]
VRSA-29	VRS2	[16]
VRSA-30	VRS3a	[16]
VRSA-31	VRS4	[16]
VRSA-32	VRS5	[16]
VRSA-33	VRSA1	[17]
VISA -1	VISA P1386	[1]
VISA -2	Mu50	[2]
VISA -3	VISA AC	[4]

VISA -4	NRS1	[5]
VISA -5	NRS19	[5]
VISA -6	NRS37	[5]
VISA -7	700699	[9]
VISA -8	VISA	[18]
VISA -9	VISA H4	[19]
VISA -10	VISA	[10]
VISA -11	hVISA	[10]
VISA -12	VISA01	[11]
VISA -13	VISA02	[11]
VISA -14	VISA03	[11]
VISA -15	VISA (1)	[12]
VISA -16	VISA SG511	[20]
VISA -17	Mu50	[20]
VISA -18	VISA CCARM 3501	[21]
VISA -19	hVISA 32679	[22]
VISA -20	hVISA 35197	[22]
VISA -21	hVISA 32681	[22]
VISA -22	VISA 22783	[22]
VISA -23	VISA 24764	[22]
VISA -24	VISA 22796	[22]
VISA -25	VISA Mu50	[23]
VISA -26	VISA KM126	[23]
VISA -27	SA137/ 93 A	[24]
VISA-28	Mu50	[25]
VISA-29	ATCC 700699	[26]
VISA-30	Mu50	[27]
VISA-31	Mu50	[28]
VISA-32	Mu50	[29]
VISA-33	NRS1	[16]
